# Generalized Anxiety Disorder and Obesity: Overlapping Neuroendocrine, Metabolic, and Behavioral Pathways

**DOI:** 10.3390/nu17172835

**Published:** 2025-08-31

**Authors:** Agnieszka Dymek, Magdalena Zielińska, Anna Englert-Bator, Katarzyna Dereń, Edyta Łuszczki

**Affiliations:** Faculty of Health Sciences and Psychology, Collegium Medicum, University of Rzeszów, 35-959 Rzeszów, Poland; mazielinska@ur.edu.pl (M.Z.); aenglert@ur.edu.pl (A.E.-B.); kderen@ur.edu.pl (K.D.); eluszczki@ur.edu.pl (E.Ł.)

**Keywords:** anxiety disorders, generalized anxiety disorder, gut microbiota, insulin resistance, mental health, obesity, sleep disturbance

## Abstract

**Background/Objectives**: Generalized anxiety disorder (GAD) is one of the most commonly diagnosed anxiety disorders in primary care. The global lifetime prevalence of GAD is estimated at 3.7%, ranging from 1.6% in low-income countries to 5.0% in high-income countries, underscoring its widespread impact. Given the frequent co-occurrence of GAD with obesity, this association has important clinical implications, particularly for screening, prevention, and treatment strategies. The aim of this review is to identify potential biological mechanisms linking obesity and GAD, summarize the current state of knowledge in this area, and highlight existing research gaps, as well as directions for future research. **Methods**: This narrative review is based on the literature published between 2015 and 2025 concerning the co-occurrence of GAD and obesity, with a focus on potential shared mechanisms including HPA axis dysregulation, chronic inflammation, oxidative stress, insulin resistance, gut–brain axis and microbiota dysbiosis, sleep disturbance, and maladaptive eating behaviors. **Results/Conclusions**: A growing body of evidence suggests an important, albeit still ambiguously defined, relationship between obesity and GAD. GAD and obesity may reinforce each other, leading to a mutually reinforcing relationship. Despite growing interest, high-quality prospective and interventional studies focusing specifically on GAD are lacking. A potentially effective therapeutic approach should be integrated and multidisciplinary, combining psychological, pharmacological, and lifestyle interventions. It may also be beneficial for clinicians to consider routine assessment of anxiety in patients with obesity and, conversely, to monitor metabolic risk in individuals with GAD. Such an approach, targeting both mental and metabolic domains, holds promise for improving outcomes.

## 1. Introduction

The interplay between mental health and physical health is a topic of increasing interest in contemporary research, offering profound implications for understanding and addressing some of the most pressing public health challenges. According to the Global Burden of Disease (GBD) 2019 estimates, age-standardized rates of mental disorders remained broadly stable between 1990 and 2019; however, the absolute number of cases and the proportion of disability-adjusted life years (DALYs) attributable to these conditions increased as a result of population growth and aging. Anxiety disorders (ADs), together with depression, are among the most frequently diagnosed mental health conditions in modern societies [1]. In the first year of the coronavirus disease 2019 (COVID-19) pandemic, the global prevalence of major depressive disorder and anxiety disorders rose markedly (by approximately 27.6% and 25.6%, respectively) [2]. Some of the observed increases in recorded diagnoses may reflect improved case detection and declining stigma, which facilitate more frequent help-seeking; in many countries, this has coincided with documented rises in the utilization of psychiatric services. In recent decades, broader public awareness and the development of initiatives promoting mental health have further encouraged individuals to seek professional support [3]. At the same time, several environmental, social, and behavioral factors may contribute to genuine increases in symptom burden; these include, for example, social determinants of health such as economic inequality and job insecurity, alongside behavioral aspects like high social media exposure, reduced physical activity, limited daylight, and poor sleep [3,4].

ADs are defined as conditions characterized by excessive fear and anxiety, accompanied by behavioral disturbances that cause significant distress or impairment in daily, social, educational, or occupational functioning. Fear represents an immediate response to a perceived present threat, whereas anxiety is oriented toward anticipation of a future threat. The specific focus of fear or anxiety distinguishes different subtypes of AD, and associated cognitive processes further shape their clinical presentation [5]. Fear, anxiety, excessive worrying, and nervous tension are characteristic symptoms of AD. They are frequently accompanied by somatic manifestations such as palpitations, dyspnea, increased muscle tension, or dizziness. However, similar symptoms may occur in stressful situations, so even their intense severity, if transient, does not clearly indicate a diagnosis of AD [6]. Diagnostic criteria for anxiety disorders are described in commonly used classification systems, including the DSM (DSM-IV, DSM-5) and the ICD (ICD-10, ICD-11). The main subtypes of AD include panic disorder, social anxiety disorder, agoraphobia, specific phobias, separation anxiety disorder (SAD), selective mutism, and generalized anxiety disorder (GAD) [7]. These disorders may co-occur and co-exist with other clinical entities such as depression, bipolar affective disorder, or obsessive–compulsive disorder [7,8].

Among these subtypes, GAD is one of the most commonly diagnosed ADs in primary care [6]. The global lifetime prevalence of GAD is estimated at 3.7%, ranging from 1.6% in low-income countries to 5.0% in high-income countries, underscoring its widespread impact [9]. According to the established definition, it is characterized by persistent and severe anxiety symptoms lasting at least six months. These symptoms can manifest as generalized anxiety and excessive worrying about various aspects of daily life, including family, work, health, or financial matters [5]. GAD is often accompanied by characteristic symptoms that include chronic fatigue, anxiety, difficulty concentrating, irritability, sleep disturbances, and increased muscle tension [6]. Two questionnaires are mainly used in the screening diagnosis of GAD: the Generalized Anxiety Disorder-7 (GAD-7) and its abbreviated version, the GAD-2. If a patient’s score on a screening tool meets the threshold indicating a probable diagnosis, this suggests the possible presence of an AD. However, it should be noted that a screening result alone is insufficient for a definitive diagnosis. A comprehensive clinical interview is required, based on standardized diagnostic criteria, including an assessment of symptom persistence and the extent to which they contribute to psychological distress and/or impair daily functioning [6]. First-line pharmacological treatment includes selective serotonin reuptake inhibitors (SSRIs) and serotonin–norepinephrine reuptake inhibitors (SNRIs) [10,11]. Regarding psychological interventions, the recommended adjunctive treatment for GAD is cognitive behavioral therapy (CBT) [12].

In parallel with the rising prevalence of psychiatric disorders, obesity rates have been steadily increasing worldwide. The World Health Organization (WHO) defines obesity as an abnormal or excessive accumulation of body fat that negatively impacts health, typically corresponding to a body mass index (BMI) ≥ 30 kg/m^2^ [13]. According to the WHO classification, obesity is further subdivided into three classes based on BMI, Class I (30.0–34.9 kg/m^2^), Class II (35.0–39.9 kg/m^2^), and Class III (≥40.0 kg/m^2^), with higher classes associated with progressively increased risk of obesity-related morbidity and mortality. Due to its scale and rapid spread, obesity is now classified as a global epidemic and represents one of the most significant public health challenges of the 21st century [13]. The primary cause of weight gain leading to obesity is a sustained positive energy balance, most commonly resulting from excessive consumption of high-energy foods. However, it is essential to emphasize that the pathophysiology of obesity is highly complex, involving numerous interactions among genetic, behavioral, environmental, economic, and sociocultural factors that contribute to energy imbalance in individuals with excessive body weight [14]. In addition, a small proportion of cases may be attributed to monogenic or syndromic forms of obesity, which require distinct diagnostic and therapeutic considerations [15].

The relationship between GAD and obesity stands out as a particularly complex and multifaceted issue intertwining psychological, biological, and behavioral dimensions. There is evidence suggesting a bidirectional relationship between excessive body weight and mental health, with anxiety driving behaviors like emotional eating and stress-induced weight gain. On the other hand, obesity exacerbates anxiety symptoms through factors including inflammation, body dissatisfaction, and social stigma. Understanding this intricate relationship is crucial for developing integrated treatment approaches that address both mental and physical health [16].

**Figure 1:**
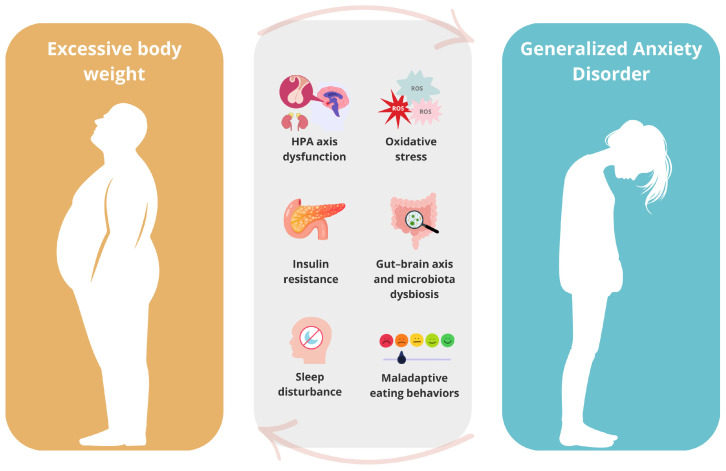
Contributing factors linking obesity and GAD. Source: Authors’ own work.

In the context of AD, several studies have reported a co-occurrence of excessive body weight and elevated anxiety levels [16,17,18,19]. A meta-analysis focusing on AD as a whole found a higher prevalence of anxiety among individuals with obesity or overweight compared to those with normal weight [20]. A similar association has been observed specifically for GAD, although the number of available studies in this area remains limited [21,22,23,24]. Potential mechanisms underlying the relationship between AD and obesity include dysfunction of the hypothalamic–pituitary–adrenal (HPA) axis [25,26], chronic inflammation [27,28], oxidative stress [29,30], insulin resistance [27,31], gut dysbiosis [32,33,34], sleep disturbances [35,36,37], and maladaptive eating behaviors [38,39] (Figure 1).

Patients diagnosed with both ADs (including GAD) and obesity represent a particularly complex clinical case that requires a multifaceted therapeutic approach. In this context, behavioral interventions—encompassing targeted dietary therapy and lifestyle modification—may serve as a key component of treatment by addressing the underlying mechanisms shared by both conditions. Moreover, such interventions may also have a preventive function by reducing the risk of developing GAD in individuals who are either healthy or living with obesity. To the best of our knowledge, no comprehensive review has yet been published that synthesizes the existing evidence on the relationship between GAD and obesity. Previous work, such as the meta-analysis by Amiri et al. [20], focused primarily on the prevalence of anxiety symptoms in individuals with obesity. In contrast, the present review aims to provide a broader perspective by identifying potential biological mechanisms linking these two conditions, summarizing the current state of knowledge in this area, and highlighting existing research gaps as well as directions for future investigation. The selection of mechanisms discussed in this review was informed by evidence demonstrating their dual relevance to both GAD and obesity. These factors are among the most frequently identified in the literature as shared biological and behavioral pathways that may mediate the bidirectional relationship between these conditions. Furthermore, they represent potentially modifiable targets for preventive and therapeutic interventions, making them particularly important from both a clinical and public health perspective.

## 2. Materials and Methods

This paper presents a narrative literature review aimed at synthesizing the current state of knowledge on the mechanisms linking anxiety disorders, with a particular focus on GAD and obesity. Due to the limited number of high-quality randomized controlled trials (RCTs) and the complex, interdisciplinary nature of the issue, a narrative review was considered the most suitable approach.

**Figure 2:**
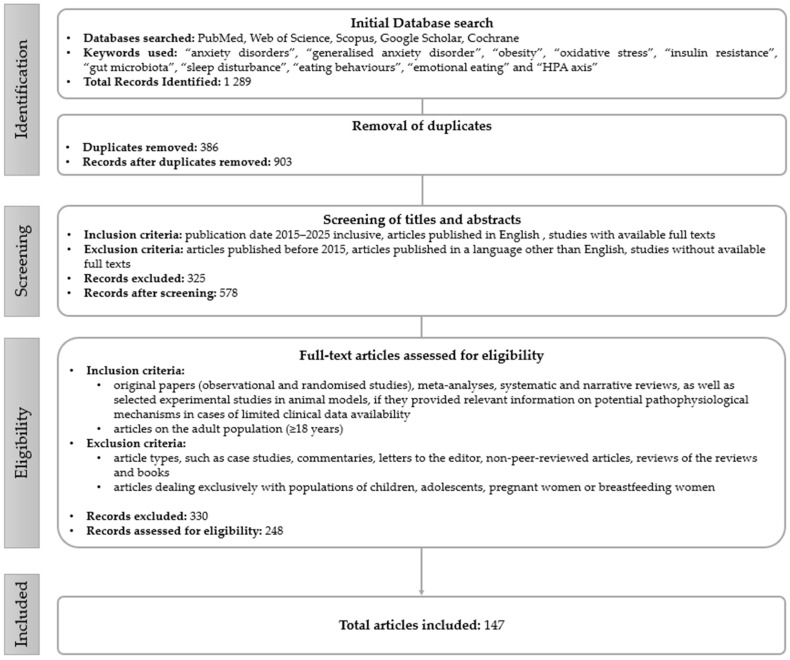
Literature search and selection flowchart for this narrative review.

The literature search was conducted in databases such as PubMed, Web of Science, Scopus, Google Scholar, and Cochrane. Combinations of keywords such as “anxiety disorders”, “generalized anxiety disorder”, “obesity”, “oxidative stress”, “insulin resistance”, “gut microbiota”, “sleep disturbance”, “eating behaviors”, “emotional eating”, and “HPA axis” were used in the search process using appropriate Boolean operators. To ensure that the data presented was up to date, the temporal scope of the publication was limited to 2015–2025. The additional literature was sourced using a cascade search strategy, which included forward and backward citation analysis. This enabled identification of relevant papers that were not included in the original database search. Although we illustrate the literature selection process in Figure 2 for transparency, this study is a narrative review rather than a formal systematic review (no quantitative meta-analysis or risk-of-bias assessment was performed). The results were organized into thematic categories that were derived inductively from recurring mechanistic pathways identified in the literature, rather than being pre-specified according to outcome measures or a quantitative threshold of included studies.

**The inclusion criteria were as follows:**

- Articles published between 2015 and 2025;
- Original papers (observational and randomized studies), meta-analyses, systematic and narrative reviews, as well as selected experimental studies in animal models, if they provided relevant information on potential pathophysiological mechanisms in cases of limited clinical data availability;
- Articles with access to the full text;
- Articles published in English;
- Articles on the adult population (≥18 years).

**The exclusion criteria included the following:**

- Articles published before 2015;
- Article types, such as case studies, commentaries, letters to the editor, non-peer-reviewed articles, reviews of the reviews, and books;
- Articles for which the full text is not accessible;
- Articles published in languages other than English;
- Articles dealing exclusively with populations of children, adolescents, pregnant women, or breastfeeding women.

Figure 2 shows the literature search process used in this narrative review.

## 3. Mechanisms Linking Anxiety Disorders and Obesity

### 3.1. HPA Axis Dysfunction

HPA axis plays a central role in mediating physiological responses to psychological stressors, thereby influencing a wide range of health outcomes. Activation of the HPA axis is a fundamental neuroendocrine response to both physical and mental stress, resulting in the release of corticotropin-releasing hormone (CRH) and adrenocorticotropic hormone (ACTH). Subsequently, cortisol is of great importance in numerous physiological processes, as chronic psychological stress can lead to overstimulation in the HPA axis [40]. According to research, such hyperactivity of the HPA axis may contribute to mental health issues. The most popular psychological disorders related to dysregulation of the HPA axis include depressive and stress-related disorders, such as GAD and post-traumatic stress disorder (PTSD) [41,42]. Studies have found that individuals with a history of trauma or chronic stress exhibit notable alterations in cortisol regulation and HPA axis functioning, which may partly reflect genetic predisposition and heritability [43].

Persistent activation of the HPA axis leads to sustained cortisol secretion, which is believed to impair emotional regulation and exacerbate stress-related symptoms [44]. Biological models of GAD often point to neurochemical imbalances, particularly the role of elevated cortisol levels caused by chronic stress, which increase appetite and promote fat storage [45]. Elevated cortisol levels are also associated with metabolic changes that increase the risk of obesity, proving the bidirectional relationship between GAD and physical health outcomes. The co-occurrence of GAD and obesity can be largely attributed to shared pathways, including both dysregulation of the HPA axis and maladaptive behaviors like emotional eating.

Early life stress, such as adverse childhood experiences (ACEs), disrupts biological systems such as the HPA axis and fosters obesogenic eating behaviors. Both of them increase the risk of anxiety in adulthood [46]. When stress disrupts the HPA axis, it leads to prolonged cortisol imbalances that can trigger anxiety and promote cravings for calorie-dense, sugary, and fatty foods. Not only does this maladaptive coping mechanism contribute to anxiety, but it also perpetuates poor dietary habits that persist into adulthood, creating a vicious cycle between anxiety and obesity.

Moreover, emotional dysregulation stemming from early life stress exacerbates maladaptive coping, such as emotional eating, compounding its long-term mental and physical health impacts. Evidence from previous studies suggests that women may be more frequently affected by early life stress due to hormonal and sociocultural factors. For example, adverse experiences are linked to increased gestational weight gain and poorer mental health outcomes during pregnancy [47]. These findings underscore the importance of integrating early interventions targeting stress management alongside dietary education to disrupt the long-term trajectory of anxiety and obesity in at-risk populations.

At the same time, emotional eating very often serves as a coping mechanism for individuals managing their anxiety. It contributes to rapid weight gain and perpetuates a cycle of psychological and physical distress. Given these shared mechanisms, interventions addressing both HPA axis dysregulation and emotional eating may be critical for disrupting the cycle. Nonetheless, existing strategies seldom adopt a fully integrated psychological and physiological framework.

### 3.2. Oxidative Stress

Oxidative stress (OS) is defined as an imbalance between the production of reactive oxygen species (ROS), including superoxide anion (O_2_^−^) and hydrogen peroxide (H_2_O_2_), and reactive nitrogen species (RNS), such as nitric oxide (NO) and peroxynitrite (ONOO^−^), and the body’s ability to neutralize them through antioxidant mechanisms [48]. ROS are generated mainly in the mitochondria during oxidative phosphorylation, but also by enzymatic systems such as nicotinamide adenine dinucleotide phosphate oxidase (NADPH oxidase), xanthine oxidase, and uncoupled nitric oxide synthase (NOS). Nitric oxide is produced primarily by nitric oxide synthases: neuronal NOS (nNOS) and endothelial NOS (eNOS) under physiological conditions, and inducible NOS (iNOS) in activated immune cells (e.g., macrophages and microglia) during inflammation. At high concentrations, molecules formed under OS conditions can damage cellular structures, leading to the degradation of DNA, lipids, and proteins [49,50]. Chronic OS often co-occurs with inflammation, and the two processes exacerbate each other [51,52]. OS has been shown to play an important role in the development of numerous chronic diseases, including obesity and AD, suggesting that these conditions share common pathophysiological mechanisms and interact with OS [53].

The relationship between obesity and OS is bidirectional: OS can result from and contribute to obesity [54]. In mitochondria, the primary site of ROS generation is the electron transport chain, particularly complexes I and III, where electron leakage to molecular oxygen produces O_2_^−^, which is then converted to H_2_O_2_ by mitochondrial superoxide dismutase (MnSOD). In the cytosol, ROS are produced mainly by enzyme systems such as NADPH oxidases (NOX family), xanthine oxidase, and uncoupled nitric oxide synthase. These distinct subcellular sources differ in their regulation and contribution to oxidative damage, but both can be upregulated in obesity and contribute to metabolic dysfunction [48,49]. The main biochemical mechanisms favoring increased ROS production in obesity include chronic low-grade inflammation in adipose tissue, mitochondrial dysfunction, and superoxide generation through the action of NADPH [55]. In addition, OS can modulate adipogenesis by affecting transcription factors dependent on oxidation–reduction imbalance, such as peroxisome proliferator γ (PPARγ), CCAAT binding protein/enhancer β (C/EBPβ), and PPARγ coactivator 1α (PGC-1α) [56,57]. Adipocytes and preadipocytes produce pro-inflammatory cytokines, such as TNF-α and IL-6, which stimulate immune cells to produce ROS and increase OS [58]. Another source of ROS in obesity may be the activation of NADPH oxidase by angiotensin II, since this enzyme is a major catalyst of free radical production [59]. Importantly, leptin–chronically elevated in individuals with obesity–activates the PI3K/PKC pathway, increasing ROS production and promoting a Th1-type inflammatory response, thereby further increasing OS and inflammation in adipose tissue [60]. Furthermore, obesity leads to excess nutrients and increased mitochondrial activity in adipocytes, resulting in increased oxidative phosphorylation and the overproduction of ROS, primarily in the form of O_2_^−^ and H_2_O_2_ [61]. Chronic mitochondrial overload promotes metabolic dysfunction, apoptosis, and disruption to the insulin signaling pathway, thereby exacerbating OS and the development of metabolic disorders [62]. ROS also affects appetite regulation by influencing proopiomelanocortin (POMC) and agouti-related protein/neuropeptide Y (AgRP/NPY) neurons in the hypothalamus [63,64]. Furthermore, a reduction in the activity of antioxidant enzymes such as superoxide dismutase (SOD), catalase (CAT), and glutathione peroxidase (GPx) has been observed in obesity [65,66]. Reduced activity of these enzymes potentiates the effect of OS and contributes to further metabolic deterioration. A study by Kanikowska et al. showed that restricting calories by 300–500 kcal per day for eight weeks led to significant improvements in the parameters of OS in patients with obesity. These improvements included a 12% increase in SOD activity and a 20% decrease in myeloperoxidase levels [67].

While the role of OS in obesity is well documented, an increasing number of studies also highlight its significant impact on AD [68]. The brain is highly vulnerable to oxidative damage due to high oxygen consumption, abundant peroxidation-prone lipids, and relatively low antioxidant defenses [69,70]. ROS can disrupt the balance of neurotransmitters, including reducing the levels of gamma-aminobutyric acid (GABA), the main inhibitory neurotransmitter in the central nervous system (CNS). This can lead to increased anxiety symptoms and excessive neuronal excitability [71]. Abnormally activated microglia are a major ROS/RNS source, releasing pro-inflammatory cytokines (e.g., TNF-α, IL-1β) and activating NF-κB, thereby worsening neuroinflammation [72,73]. NADPH oxidase overactivity in microglia is linked to neuronal damage in AD [74,75]. Chronic activation of the HPA axis during psychological stress raises glucocorticoid levels, increasing ROS production and causing oxidative injury to key brain areas like the hippocampus, amygdala, and prefrontal cortex [76,77,78]. Elevated levels of malondialdehyde (MDA), a lipid peroxidation marker, have been observed in both obesity and GAD [79]. Patients with newly diagnosed GAD also show increased 8-hydroxy-2′-deoxyguanosine (8-OHdG), indicating OS-induced DNA damage, even without changes in total oxidative or antioxidant capacity [80]. Importantly, oxidative damage in these brain regions can contribute to progressive neurodegeneration, which may co-exist with anxiety symptoms and further impair cognitive and emotional regulation [81,82].

Both obesity and AD share overlapping mechanisms that mutually exacerbate metabolic, inflammatory, and neuropsychiatric disturbances. Chronic low-grade inflammation in adipose tissue and the CNS promotes ROS overproduction by adipocytes, microglia, and immune cells [72,73]. Excessive ROS, driven by NADPH oxidase and mitochondrial dysfunction, contributes to lipogenesis, insulin resistance, and neurotransmitter imbalances [74,75], particularly through mitochondrial ROS in hypothalamic neurons, which affect appetite, mood, and stress responses [83]. Reduced activity of antioxidant enzymes (SOD, CAT, GPx) increases susceptibility to oxidative damage [66,70,84], while HPA axis overactivity and chronic cortisol elevation further amplify oxidative, metabolic, and emotional disturbances [76,85].

### 3.3. Insulin Resistance

Insulin is a hormone that is secreted by beta cells in the pancreas when blood glucose levels increase. After binding to and activating insulin receptors, it initiates a series of metabolic processes that enable fat, muscle, and liver cells to take up and utilize glucose [86]. In addition to regulating glucose levels in peripheral tissues, insulin also affects the CNS. Studies have shown that insulin and its receptors are present in almost all types of brain cells [86]. The effects of insulin in the CNS depend on the specific area of the brain. For instance, insulin binds to its receptors in the arcuate nucleus of the hypothalamus to regulate food intake and weight control. An important difference between peripheral and central glucose utilization under the influence of insulin is that, in the brain, glucose serves not only as an energy source but also plays a key role in enhancing cognitive function and supporting neuroplasticity [87].

IR is a complex metabolic condition characterized by reduced sensitivity of cells and tissues to insulin [88]. The relationship between IR and obesity is well established, with visceral fat playing a key role by promoting chronic low-grade inflammation and increased secretion of pro-inflammatory cytokines such as TNF-α and IL-6 [89,90]. Inflammation in individuals with obesity is one of the major factors disrupting normal insulin signaling. Studies indicate that inflammation may contribute to the development of IR by inhibiting the activity of insulin receptor substrate 1 (IRS-1) in adipose and skeletal muscle tissue. In other cases, inflammation promotes IR by limiting the activity of PPARγ, triggering excessive release of free fatty acids, and activating the NLRP3 inflammasome [88]. Barber et al. also suggest that obesity and IR may be linked through other complex mechanisms, such as mitochondrial dysfunction leading to the overproduction of reactive oxygen species, intestinal dysbiosis, or remodeling of the adipose tissue extracellular matrix [91]. Studies indicate that peripheral IR can lead to reduced insulin receptor activity in brain tissue, thereby limiting insulin bioavailability in brain structures. This may cause dysfunction in brain regions such as the frontal lobe, which is critical for emotion regulation, thereby increasing susceptibility to anxiety disorders [87,92]. Additionally, IR affects the functioning of the hippocampus, leading to hyperactivity of the HPA axis [93]. As a consequence, disturbances occur in the circadian rhythm of cortisol secretion and the balance between mineralocorticoids and glucocorticoids, which negatively impact the homeostasis of the serotonergic system, particularly serotonin (5-HT) levels. Dysfunction of this system appears to be an important factor in the pathogenesis of anxiety disorders [94,95].

Conversely, there is evidence to suggest that anxiety may increase the excitability of the HPA axis, resulting in elevated cortisol levels [95,96]. Chronic elevation of this hormone inhibits insulin-mediated glucose uptake, leading to hyperglycemia. Cortisol also exerts a potent lipogenic effect, and excessive production may contribute to the development of obesity and dyslipidemia [93,97]. Furthermore, anxiety amplifies the inflammatory response, increasing the production of pro-inflammatory factors such as IL-6 and TNF-α. The presence of these cytokines represents a significant risk factor for IR and may promote the onset of metabolic syndrome, of which obesity is a central component [27]. Therefore, it may be assumed that IR, as a mechanism mediating the relationship between anxiety disorders and obesity, operates in a bidirectional manner.

Current scientific research highlights the existence of complex interrelationships between IR, obesity, and AD. One study investigated whether symptoms of social anxiety moderate the relationship between obesity and inflammation, as well as between obesity and IR [98]. Moderation analysis revealed that the association between larger waist circumference and higher levels of inflammatory markers and insulin resistance was significantly stronger among individuals with elevated social anxiety. While a similar pattern was observed in both low and high social anxiety groups, the effect was approximately 1.5 times greater in those with higher anxiety levels. A study by Díaz-Carias et al. [99] reported a significant correlation between anxiety, insulin levels, waist circumference, and BMI. However, the number of studies directly examining the relationship between GAD and these metabolic factors remains limited. A recently published study assessing the prevalence of IR and metabolic syndrome (MetS) in patients with GAD revealed a high incidence of these conditions in the study group, although no significant correlation was found with the severity of anxiety symptoms [100]. IR was diagnosed in nearly 40% of participants, while approximately 35% met the diagnostic criteria for MetS. These findings suggest that individuals with GAD may be at increased risk of developing metabolic disorders [100]. A 2023 meta-analysis further confirmed the association between anxiety and MetS [101]. The authors emphasized the need to systematically monitor metabolic parameters in individuals treated for AD and, conversely, to recognize the heightened risk of AD in patients with MetS. While this study referred to AD in general, its findings remain relevant for understanding metabolic risk in populations with GAD.

In addition to insulin resistance, other hormonal pathways may also contribute to the interplay between obesity and anxiety disorders. In particular, leptin is a key regulator of appetite and energy balance. Although leptin deficiency represents a rare monogenic cause of severe early-onset obesity, common forms of obesity are usually associated with elevated circulating leptin levels and resistance to its anorexigenic and energy expenditure-stimulating effects [102]. Several factors contribute to hyperleptinemia and leptin resistance, including excessive energy intake, increased adipose tissue mass, overfeeding, hyperinsulinemia, estrogens, and pro-inflammatory cytokines [103]. Physiologically, leptin suppresses food intake through hypothalamic pathways involving proopiomelanocortin (POMC) neurons and enhances energy expenditure, partly via the activation of thermogenic processes in skeletal muscle and adipose tissue [104]. Beyond metabolic regulation, increasing evidence indicates that leptin also exerts neurotrophic effects, supporting neurogenesis, axonal growth, synaptogenesis, and neuroprotection [105]. Dysregulated leptin signaling has been associated not only with overeating and weight gain but also with altered stress responses and mood disturbances, which may contribute to the increased susceptibility to anxiety disorders observed in obesity [104,106]. Importantly, impaired leptin signaling and its effects on the hippocampus may also adversely influence reward system functioning, thereby promoting addiction-like behaviors such as overeating [105].

### 3.4. Gut–Brain Axis and Microbiota Dysbiosis

The gut microbiota constitutes a complex ecosystem of microorganisms—including bacteria, viruses, fungi, and archaea—that inhabit the human digestive tract. Its composition is influenced by a range of factors, including genetics, lifestyle, diet, health status, age, and geographical location [107]. Under physiological conditions, the microbiota performs a variety of beneficial functions for the host, such as producing bioactive compounds (e.g., vitamins and short-chain fatty acids (SCFAs)), regulating appetite and energy expenditure [107,108,109]. Importantly, the gut microbiota also plays a role in modulating immune responses and inflammatory processes, thereby underscoring its systemic relevance beyond the gastrointestinal tract [110]. In addition, intestinal microorganisms have been reported to modulate the availability and activity of neurotransmitters, thereby influencing central nervous system function through the gut–brain axis. This axis represents a complex bidirectional communication network that integrates the central and autonomic nervous systems, the HPA axis, the sympathoadrenal system, the immune system, and the gut microbiota. By regulating fundamental intestinal functions, it provides a framework for the exchange of neurotransmitters and signaling molecules between the gut and the brain [111]. Alterations in the composition and abundance of intestinal bacteria—a condition referred to as intestinal dysbiosis—have been linked to a range of diseases, including obesity and mental health disorders [112].

Numerous studies have shown that individuals with obesity exhibit reduced gut bacterial diversity and altered relative abundance of specific microbial groups compared to healthy individuals [113,114]. Dysbiosis in individuals with obesity promotes increased appetite, more efficient energy extraction and storage in the form of adipose tissue, and disturbances in bile acid metabolism, and contributes to systemic inflammation through the production of lipopolysaccharide (LPS), further exacerbating the disease state [115]. Research suggests that two major bacterial phyla—Firmicutes and Bacteroidetes—may serve as potential biomarkers of obesity due to their central role in human metabolism [115]. In numerous studies, particularly those involving animal models, a gut microbiota profile characteristic of obesity has been reported, typically involving an increased abundance of Firmicutes and a concurrent reduction in Bacteroidetes. However, findings from human studies remain inconsistent and inconclusive [112].

On the other hand, gut microorganisms play an important role in the functioning of the nervous system via the gut–brain axis and may influence the risk of developing anxiety disorders [116]. One potential mechanism involves the modulation of neurotransmitter pathways, including serotonin, dopamine, and GABA, as well as their precursors such as tryptophan, which are crucial in regulating mood and anxiety responses [117,118,119]. Gut microbes may, for instance, modulate the kynurenine pathway, limiting the conversion of tryptophan into serotonin. This can lead to serotonin depletion and an increased risk of anxiety and depression [120,121]. Gut dysbiosis also contributes to increased intestinal permeability, partly by reducing the abundance of SCFA-producing bacteria, which are essential for maintaining barrier integrity. A weakened intestinal barrier facilitates the translocation of pathogenic bacteria and their metabolites, including LPS, into lymph nodes and the bloodstream, thereby amplifying immune responses [116,122]. The resulting inflammation can affect the CNS through both the blood–brain barrier and vagus nerve activation [116]. Chronic stimulation of these mechanisms disrupts the functioning of the HPA axis, leading to elevated cortisol levels [116] and an increased risk of anxiety disorders [123]. Brain-derived neurotrophic factor (BDNF)—a polypeptide growth factor essential for neurogenesis and neuroplasticity—also plays a key role in this context. Higher levels of BDNF are associated with better mental health and lower vulnerability to anxiety disorders. The gut microbiota can modulate BDNF levels, and under dysbiotic conditions, its availability may be reduced due to excessive HPA axis activation, potentially increasing the risk of anxiety [124].

Available human studies indicate an altered composition of the gut microbiota in patients with GAD compared to healthy individuals [125,126,127,128]. Jiang et al. observed dysbiosis in the gut microbiota of individuals with GAD, characterized by a reduced abundance of SCFA-producing bacteria such as *Lachnospira*, *Sutterella*, *Butyricicoccus*, *Faecalibacterium*, and *Eubacterium rectale*, alongside an excessive proliferation of *Escherichia*-*Shigella*, *Fusobacterium*, and *Ruminococcus gnavus* [125]. At the phylum level, a marked increase in Bacteroidetes spp. relative to Firmicutes spp. was noted. Importantly, these unfavorable changes in the bacterial population persisted even during periods of disease remission. The authors suggest that the gut microbiota may serve as a potential target for both preventive and therapeutic interventions in GAD. Similar conclusions regarding the role of the microbiota in the development of GAD and anxiety disorders have been drawn by other researchers [126,127]. Cheng et al. conducted a study analyzing the relationship between gut microbiota and GAD, further considering inflammatory markers and *BDNF* gene polymorphisms [128]. The researchers found that patients with GAD exhibited elevated levels of TNF-α, IL-4, IL-10, and IgG. Microbiome analysis revealed an increased abundance of *Paraprevotella*, *Euryarchaeota*, *Caldivirga*, *Porphyromonadaceae*, and *Desulfovibrionales* in this group, whereas healthy individuals showed a predominance of *Lactobacillus*, *Vagococcus*, *Barnesiella*, and *Paludibacter*. Moreover, the distribution of alleles, genotypes, and haplotypes of the BDNF gene differed between patients and the control group [128].

A recently published systematic review and meta-analysis suggests a positive correlation between mood disorders and obesity, with the gut microbiota playing a significant role in both conditions [112]. However, studies involving human participants that directly investigate the relationship between excessive body weight and GAD or other anxiety disorders in the context of gut microbiota remain scarce. Given that intestinal dysbiosis is commonly observed in both individuals with obesity and those with GAD, it appears that the gut microbiota may represent an important target for therapeutic and preventive interventions.

### 3.5. Sleep Disturbance

Approximately 20–35% of adults regularly experience insufficient sleep, defined as less than 6 h per day, despite current recommendations indicating an optimal duration of 7 to 9 h per day for adults [129,130]. Inadequate sleep duration, in addition to increasing the risk of cardiometabolic disorders [131], is also significantly associated with excessive body weight [132,133,134] and heightened anxiety symptoms [135,136,137].

A systematic review and meta-analysis showed that short sleep (<6 h) is linked to an 8% higher risk of abdominal obesity, whereas long sleep (≥8–9 h) showed no such association [133]. Several mechanisms have been proposed to explain the link between sleep deprivation and obesity, including hormonal imbalances, particularly involving leptin and ghrelin [138], overactivation of the HPA axis resulting in increased cortisol secretion [132,139], elevated levels of pro-inflammatory cytokines, and unfavorable lifestyle changes that promote a positive energy balance. Sleep duration may influence the concentration of appetite-regulating hormones—leptin (a satiety hormone) and ghrelin (a hunger hormone). Both act on the arcuate nucleus (ARC) of the hypothalamus, which plays a key role in appetite control and energy expenditure regulation. Leptin exerts an anorexigenic effect—it reduces appetite, increases energy expenditure, and inhibits lipogenesis. Ghrelin, on the other hand, is orexigenic, stimulating food intake [140]. Although research findings in this area remain inconsistent, some studies suggest that short sleep duration may lower leptin levels and increase ghrelin levels, potentially leading to increased appetite and weight gain [141,142]. However, the most recent meta-analysis indicates that sleep deprivation does not produce clear and consistent effects on these hormones, highlighting the need for further well-designed studies [143]. Short or fragmented sleep is also associated with elevated levels of inflammatory markers, particularly IL-6 and high-sensitivity C-reactive protein (hs-CRP). Sleep deprivation may exacerbate inflammation further through HPA axis activation and elevated cortisol levels, thereby promoting visceral fat accumulation [139]. A meta-analysis demonstrated that partial sleep restriction over several consecutive nights (mean 8.3 ± 5.6 nights, reduced to approximately 4.5 h per night) significantly increased CRP and IL-6 levels compared to participants with normal sleep duration [144]. Moreover, poor sleep quality has been shown to positively correlate with inflammatory marker levels in adults with obesity [145]. This relationship may be bidirectional—weight loss has been associated with both improved sleep quality and reductions in inflammatory markers, as confirmed in patients with chronic insomnia [146]. Increasing attention has also been paid to the role of chronotype. In individuals with obesity and an evening chronotype, higher levels of inflammatory markers (including IL-1β, IL-8, bFGF, MCP-1, and MIP-1β) have been observed in visceral adipose tissue compared to morning chronotypes, despite similar BMI and body composition [147]. Short sleep duration promotes unhealthy dietary behaviors by increasing the frequency of eating, especially high-calorie, processed foods rich in fats and sugars. Sleep deprivation enhances reward-related brain activity, disrupts appetite regulation, and extends wakefulness, which together raise total energy intake and encourage impulsive late-night eating [148,149].

Anxiety disorders (AD) have also been strongly linked to sleep disturbances. Data from a two-sample Mendelian randomization analysis indicate that individuals with sleep disorders have nearly double the risk of developing anxiety compared to those without such disorders (OR = 1.89; 95% CI: 1.43–2.48). Conversely, the presence of AD increases the likelihood of experiencing sleep problems by approximately 20% (OR = 1.20; 95% CI: 1.03–1.39) [150]. Sleep disturbances in individuals with heightened anxiety are multifactorial and result from dysregulation of neurotransmitter systems, hormonal pathways, brain structures, and the autonomic nervous system [137]. A key mechanism involves chronic hyperarousal, which hinders the transition to sleep. This is associated with an imbalance between increased glutamatergic activity and diminished GABAergic transmission. GABA deficiency impairs the function of sleep-promoting structures, particularly the ventrolateral preoptic nucleus (VLPO), leading to overactivation of wakefulness-promoting systems [137,151]. In anxious states, hyperactivity of the amygdala and weakened functional connectivity with the medial prefrontal cortex (mPFC) impair emotional regulation and promote ruminative thinking, further hindering sleep initiation [135]. Dysregulation of the HPA axis contributes to elevated evening cortisol levels, which disrupt sleep architecture by delaying sleep onset, reducing slow-wave sleep, and increasing night-time awakenings [152,153].

Disrupted sleep is not merely a consequence of anxiety but actively contributes to its exacerbation, creating a self-perpetuating feedback loop. Under conditions of sleep deprivation, amygdala reactivity to emotional stimuli increases, while top-down control exerted by the medial prefrontal cortex (mPFC) is diminished. This imbalance leads to heightened anxiety and impaired threat processing [154,155]. Furthermore, a reduction in GABA levels—particularly within the prefrontal cortex and cingulate gyrus—limits the brain’s ability to inhibit excessive neuronal activation, thereby intensifying anxious symptoms [156,157,158]. Sleep deprivation also elevates the concentration of pro-inflammatory cytokines (CRP, IL-6, TNF-α), which, through microglial activation, induce neuroinflammatory changes in brain regions involved in emotional regulation, such as the amygdala, hippocampus, and prefrontal cortex [159,160,161]. Inadequate or irregular sleep disrupts cortisol and melatonin secretion, leading to chronic hyperarousal and reduced adaptability, resulting in emotional instability, impulsivity, and impaired cognition [162,163,164,165].

The reciprocal reinforcement of anxiety mechanisms and sleep disturbances contributes to the development of a vicious cycle, particularly evident in individuals with GAD, where chronic worry and heightened arousal co-occur with insomnia and disrupted sleep architecture [137].

Despite existing evidence suggesting interrelationships among sleep disturbances, obesity, and AD, there remains a lack of studies, especially in the context of GAD, that examine these three factors simultaneously. A recent cross-sectional study examined the link between sleep quality and physical and mental health in individuals with overweight, obesity, and normal weight [166]. In both groups, a statistically significant association was found between poor sleep quality and greater severity of anxiety symptoms, as measured by the GAD-7 questionnaire. This effect was slightly stronger in the overweight or obese group (β = 0.71; 95% CI: 0.62–0.79) than among participants with normal body weight (β = 0.67; 95% CI: 0.57–0.76), even after adjusting for confounding variables. In a study by Alhusseini et al., a significant relationship was also demonstrated between obesity, sleep quality, and the severity of anxiety symptoms [167]. Participants with overweight and obesity scored significantly higher on the Pittsburgh Sleep Quality Index (PSQI) compared to those who were underweight (mean scores: 8.6 ± 3.4; 8.9 ± 3.6 vs. 7.5 ± 3.0, respectively; *p* = 0.001). Moreover, more severe anxiety symptoms, assessed using the GAD-7, were associated with poorer sleep quality—individuals with moderate and severe anxiety attained higher PSQI scores (9.7 ± 3.7 and 10.4 ± 3.6, respectively) compared to those with minimal anxiety levels (6.8 ± 2.7; *p* < 0.001). Similar associations were observed in another cross-sectional study involving 130 adults with overweight or obesity, in which higher levels of anxiety were more frequently linked with poorer self-reported sleep quality (r ≈ 0.28; *p* = 0.0011), with the association particularly pronounced among participants under the age of 45 [168].

### 3.6. Maladaptive Eating Behaviors

There are significant links between AD, including GAD, and abnormal eating behaviors [169]. Although anxiety can affect appetite in different ways, the tendency to overeat is most commonly observed in the context of GAD, increasing the risk of obesity [170,171]. The literature particularly highlights the role of three maladaptive eating behaviors as key mechanisms mediating this relationship: emotional eating (EE), uncontrolled eating (UE), and cognitive restraint (CR) [169,171,172].

EE, which is defined as eating in response to emotions rather than actual physiological hunger, plays a key role in anxiety management [169]. A review by Dakanalis et al. highlighted that EE is significantly associated with increased anxiety, stress, and increased risk of overweight and obesity, acting as a coping strategy for negative affect [173]. Individuals experiencing heightened anxiety symptoms often reach for high-energy, palatable foods to relieve tension, which is achieved through, among other things, activation of the reward system (mainly dopaminergic pathways) [173,174]. Although the relief effect is short-lived, this behavior can persist, resulting in weight gain and exacerbated anxiety symptoms. This creates a vicious cycle involving emotions and neurophysiology [175]. This is influenced by factors such as difficulties in regulating emotions, impaired recognition of hunger and satiety signals, or the use of eating as a strategy to avoid or suppress unpleasant internal sensations [176,177,178]. A study by Fonseca et al. found that in women with GAD, EE severity correlates with increased worry, avoidance of internal sensations, difficulty regulating emotions, low levels of self-compassion, and reduced attentiveness [179]. Women are particularly susceptible to such mechanisms, which may be due to both the higher prevalence of AD in this group and sociocultural pressures related to appearance [175,179]. Importantly, EE is also a risk factor for the development of eating disorders, particularly binge eating disorder (BED) [180]. A systematic review by Arexis et al. revealed significant similarities between EE and BED [178]. Both individuals experiencing EE and patients with BED exhibited difficulties with emotion regulation and impulse inhibition, suggesting a potential continuum between the two conditions.

UE refers to the loss of control over the amount of food consumed in response to various internal and external stimuli [169]. UE is underpinned by neurobiological dysfunctions in reward processing, particularly in the activity of the nucleus accumbens, amygdala, and prefrontal cortex. These dysfunctions increase susceptibility to compulsive eating despite awareness of the negative consequences [181]. Furthermore, impaired self-regulation, which is often observed in individuals with GAD, is significantly associated with the severity of UE and a higher BMI [182]. Both anxiety and UE are associated with higher neuroticism and lower self-efficacy, which further increases risk [169,170]. Anxiety and stress affect hormonal regulation, including the function of leptin and ghrelin, thereby disrupting satiety mechanisms and increasing appetite [183,184]. Importantly, growing evidence suggests that neuropeptide Y (NPY), which is activated during states of hunger, increases appetite and alleviates anxiety symptoms, as well as promoting the extinction of fear responses, which may have adaptive significance [185]. Conversely, satiety neuropeptides such as corticotropin-releasing hormone (CRH) and cholecystokinin (CCK) have anorexigenic and anxiogenic effects, respectively, indicating the presence of shared neurobiological pathways involved in regulating both emotion and energy homeostasis. A study by Witaszek et al. found that a higher BMI in women was associated with greater EE and UE severity, alongside a reduction in CR. Women with GAD exhibited higher levels of inappropriate eating behaviors [171].

CR refers to the cognitive effort an individual makes to restrict their food intake to control their body mass weight [169]. While it could theoretically serve a protective function against obesity, in reality, it is often associated with social pressure and low self-esteem, which limits its effectiveness [186,187]. Research indicates that people with obesity and heightened anxiety symptoms are more likely to experience difficulties with EE and UE, while showing lower levels of CR and self-efficacy in regulating eating behavior. Importantly, the severity of these difficulties increases in proportion to the level of anxiety symptoms [170]. According to Hussenoeder et al., anxiety acts as an internal confounder that intensifies disinhibition (overeating) and temporarily weakens the impact of CR [169]. Furthermore, Hussenoeder et al. observed that CR correlates more strongly with stable personality traits than with anxiety as an emotional state. After accounting for personality in the regression model, anxiety was found to be a non-significant predictor of eating behavior [169]. In other words, their findings suggest that personality-driven impulsivity and neuroticism underlie the link between anxiety and UE, rather than anxiety per se once personality is controlled. These mechanisms are partly explained by disturbances in the HPA axis, elevated cortisol levels, and dysregulation of systems responsible for reward processing and impulsivity control [188]. Disruption to the gut–brain axis may also contribute, as it affects the metabolism of neurotransmitters such as serotonin and GABA, and influences stress resistance, impulsivity, and self-regulation [189,190]. In summary, maladaptive eating behaviors, particularly EE and UE, appear to be a behavioral link between GAD and obesity. These behaviors not only contribute to weight gain but also interact with biological and psychological mechanisms that may exacerbate anxiety symptoms.

Table 1 concisely summarizes the key overlapping mechanistic pathways that link GAD and obesity, highlighting representative findings and references.

## 4. Limitations and Future Research

Despite its comprehensive coverage of the topic and consideration of numerous biological, psychological, and behavioral mechanisms, this narrative review is subject to certain limitations that must be taken into account when interpreting its conclusions. Most of the reviewed studies are observational, which restricts the ability to draw causal conclusions. The identified relationships between GAD and obesity may only be correlational, and their direction and mechanisms require verification through prospective and experimental studies. Secondly, many of the studies included in this review do not distinguish GAD as a separate diagnostic entity; instead, they analyze it within the context of a general anxiety disorder spectrum or subclinical symptoms. Such ambiguity can affect the consistency and precision with which the results are interpreted. Another limitation is the way anxiety disorders are assessed, which relies mainly on self-report tools (e.g., the GAD-7 questionnaire) and less frequently on specialist diagnoses based on clinical history. A significant barrier to definitive conclusions is the limited number of studies that have examined the co-occurrence of GAD and obesity directly. Many papers focus on anxiety in general rather than GAD specifically, which can lead to overgeneralizations. In addition, methodological heterogeneity makes it difficult to compare results. This includes differences in the definitions and classifications of anxiety disorders (e.g., different anxiety rating scales and varying diagnostic criteria), as well as the measures used to assess obesity (e.g., BMI versus body composition analysis). Such heterogeneity limits the ability to conduct consistent data analysis and synthesis. It should also be noted that narrative reviews have inherent methodological limitations. Despite using a systematic approach to select the literature, there is still a risk of subjective selection and interpretation of the data. The absence of a formal assessment of the quality of the studies included and of a meta-analysis means that the conclusions are descriptive rather than quantitative. Despite these limitations, this narrative review provides a comprehensive compilation of current knowledge regarding the potential mechanisms linking GAD and obesity and identified important research gaps. It can serve as a valuable foundation for future systematic, experimental, and clinical studies aimed at more clearly defining causality and the effectiveness of potential therapeutic interventions.

In light of the findings to date and the identified research gaps, future research should focus on several key areas. Firstly, longitudinal studies are required to track changes over time and establish the nature and strength of the relationship between GAD and obesity. At the same time, intervention studies evaluating the effectiveness of comprehensive therapies combining lifestyle modifications (diet and physical activity), pharmacotherapy, and psychological interventions should be conducted. Another important step is to delineate GAD as a distinct diagnostic entity using standardized, validated diagnostic tools and clinical assessments. Future work should also involve a deeper analysis of the mediating mechanisms that may explain the co-occurrence of GAD and obesity, such as deregulation of the HPA axis, chronic inflammation, oxidative stress, gut dysbiosis, sleep disorders, and IR. Gender, age, and hormonal differences, which may affect susceptibility, the clinical presentation, and treatment efficacy of both disorders, should also be taken into account. The role of cultural and social factors such as body stigma, appearance norms, self-image, and socioeconomic status is also of particular importance, as these factors may modulate the association between GAD and obesity. Standardization of research tools is also needed, in terms of both mental diagnosis and obesity assessment (e.g., BMI vs. body composition), to allow for results from different studies to be compared. Furthermore, more research is required into the impact of GAD pharmacotherapy, particularly SSRIs and SNRIs, on metabolic parameters and body weight. Notably, several antidepressant medications have been associated with weight change, which may influence long-term treatment outcomes and patient adherence [191]. Furthermore, there is a need to improve our understanding of the lifestyles of people with GAD, given the lack of reliable quantitative and qualitative research on their health behaviors, including diet, physical activity, smoking, and alcohol consumption. Research into eating behaviors, especially EE and UE, which may mediate the relationship between GAD and obesity and be a target for interdisciplinary interventions, is also worthwhile. The influence of metabolic and epigenetic programming on the development of GAD and obesity, including the impact of prenatal and early childhood factors, is also a promising area of research. Such studies can reveal common molecular mechanisms and inform preventive strategies. The development and empirical validation of integrated therapeutic programs combining elements of psychotherapy (e.g., CBT), nutrition education, dietary therapy, and emotion regulation methods (e.g., mindfulness and mindful eating) that are tailored to the specific needs of patients with GAD and obesity also deserve special attention. In addition to established approaches, novel interventions are being explored. Beyond standard behavioral and pharmacological treatments, emerging strategies may offer dual benefits for obesity and psychiatric symptoms. In particular, GLP-1 receptor agonists, widely used for diabetes and obesity, have been proposed to improve psychiatric outcomes as well [192]. However, recent large real-world data indicated an increased risk of depression, anxiety, and suicidal behavior in patients with obesity treated with liraglutide or semaglutide [193]. These contrasting findings highlight both the promise and the uncertainty of this approach, underscoring the need for prospective, well-controlled trials to clarify the psychiatric safety and potential benefits of GLP-1 RA therapy [194].

## 5. Conclusions

A growing body of evidence points to an important, though still not fully understood, relationship between obesity and generalized anxiety disorder (GAD). The pathophysiological mechanisms underlying this relationship remain ambiguous, although growing evidence points to the involvement of common biological pathways, including HPA axis dysregulation, chronic inflammation, oxidative stress, insulin resistance, gut–brain axis and microbiota dysbiosis, sleep disturbance, and maladaptive eating behaviors. It has been suggested that GAD and obesity may exacerbate each other, creating a mutually reinforcing relationship in which chronic anxiety promotes maladaptive behaviors (such as overeating, avoidance of physical activity, and sleep disturbances), which in turn worsen metabolic status and mental health. Despite increasing interest in this topic, there is still a lack of studies of sufficient methodological quality, particularly those focusing specifically on GAD.

In light of existing knowledge, a potentially effective therapeutic approach to patients with simultaneous GAD and obesity may be integrated and multidisciplinary. It requires a combination of psychological interventions, pharmacological interventions, and lifestyle interventions. It may also be beneficial for clinicians to consider routine assessment of anxiety in patients with obesity and, conversely, to monitor metabolic risk in individuals with GAD. Addressing co-occurring sleep problems and emotional eating behaviors could further support both mental and physical health. Ultimately, such an approach, targeting both mental and metabolic domains, holds promise for improving outcomes. Nonetheless, further prospective and interventional studies are needed before any definitive clinical recommendations can be established.

## Figures and Tables

**Table 1:** Summary of key overlapping mechanisms between GAD and obesity.

Mechanistic Pathways	Key Findings Linking GAD and Obesity	Representative References	Estimated Number of Studies (2015–2025)
HPA Axis Dysfunction	Chronic and early-life stress dysregulate the HPA axis (↑CRH/ACTH → sustained cortisol), impairing emotion regulation and heightening anxiety. Elevated cortisol increases appetite, promotes fat storage and metabolic dysregulation, raising obesity risk. Adverse childhood experiences further disrupt HPA function and foster obesogenic eating; emotional eating often serves as a coping mechanism, coupling HPA hyperactivity with weight gain and creating a self-reinforcing GAD and obesity cycle.	[40,44,46]	~10
Oxidative Stress	Oxidative stress and obesity-related low-grade inflammation (↑IL-6, TNF-α) disrupt neurotransmitter balance (↓serotonin, altered GABA), impair neural function, and promote anxiety. Shared mechanisms include chronic inflammation, mitochondrial dysfunction, NADPH oxidase activity, and reduced antioxidant defenses, leading to ROS overproduction, oxidative brain damage, appetite dysregulation, and a mutually reinforcing relationship of metabolic and neuropsychiatric disturbances.	[48,51,54,68,79,84]	~35
Insulin Resistance	IR is closely associated with visceral obesity, where chronic low-grade inflammation (↑IL-6, TNF-α) disrupts insulin signaling. Reduced insulin action in the CNS may affect the frontal lobe and hippocampus, leading to HPA axis hyperactivity, cortisol dysregulation, and serotonergic imbalance, which could increase susceptibility to GAD. Conversely, chronic anxiety stimulates HPA axis overactivity and persistent cortisol release, which impair insulin-mediated glucose uptake, promote lipogenesis, dyslipidemia, and visceral fat accumulation, and further amplify inflammation.	[88,91,93,97,99]	~20
Gut–Brain Axis and Microbiota Dysbiosis	Gut dysbiosis (↓diversity, altered Firmicutes/Bacteroidetes ratio, ↓SCFA-producing bacteria) is observed in both obesity and GAD. In obesity, it enhances energy harvest, fat storage, bile acid disturbances, and systemic inflammation via LPS translocation. In GAD, dysbiosis involves reduced beneficial taxa (e.g., *Faecalibacterium*, *Lachnospira*) and overgrowth of pathogenic bacteria (e.g., *Escherichia/Shigella*, *Fusobacterium*), impairing neurotransmitter pathways (serotonin, dopamine, GABA), increasing intestinal permeability, and reducing BDNF levels. These changes activate immune responses and HPA axis dysregulation, creating a bidirectional gut–brain feedback loop that exacerbates both metabolic and anxiety symptoms.	[112,115,116,117,118,119,126,127]	~25
Sleep Disturbance	Chronic insufficient or poor-quality sleep (<6 h/day or fragmented sleep) is associated with both obesity and GAD. In obesity, short sleep promotes hormonal imbalances (↓leptin, ↑ghrelin), HPA axis overactivation with elevated evening cortisol, systemic inflammation (↑IL-6, CRP, TNF-α), and increased intake of energy-dense foods. In GAD, sleep disturbances stem from chronic hyperarousal, glutamatergic overactivity, reduced GABAergic transmission, amygdala hyperactivity, and impaired mPFC regulation. Sleep deprivation further heightens emotional reactivity, neuroinflammation, and anxiety symptoms, contributing to metabolic and psychological dysregulation.	[132,135,137,139,150,166]	~35
Maladaptive eating Behaviors	GAD is strongly associated with maladaptive eating behaviors, especially EE and UE, driven by emotion regulation deficits, altered reward processing, hormonal/neuropeptide imbalances (e.g., leptin, ghrelin, NPY, CRH), and heightened impulsivity. These behaviors temporarily relieve stress via dopamine pathways but reduce CR and impair appetite regulation, promoting weight gain and reinforcing anxiety long-term. Stable traits like neuroticism may underlie both anxiety and disinhibited eating, suggesting that interventions improving emotion regulation and self-control (e.g., mindfulness) can benefit both weight and anxiety outcomes.	[169,170,171,173,179]	~25

↑ = increase; ↓ = decrease.

## Data Availability

Not applicable.
